# Enhancing endoscopic foraminal decompression in adult isthmic spondylolisthesis: the potential influence of lateral recess isthmic spur and our case series of an innovative craniocaudal interlaminar approach via unilateral biportal endoscopic spinal surgery

**DOI:** 10.1186/s12891-023-06544-1

**Published:** 2023-05-27

**Authors:** Tsung-Mu Wu, Moon-Chan Kim, Jin-Ho Hwang, Dae-Jung Choi

**Affiliations:** 1 Orthopedic Department, Chi-Mei Medical Center, No.901, Zhonghua Rd., Yongkang Dist., Tainan City, Taiwan (R.O.C.); 2 Spine Center, Himnaera Hospital, 85, Boemil-Ro, Dong-Gu, Busan, Korea

**Keywords:** Adult isthmic spondylolisthesis, Lumbar, Foraminal stenosis, Decompression, Endoscopic spine surgery

## Abstract

**Background:**

The NASS guideline cannot recommend any of the surgical treatment options toward adult isthmic spondylolisthesis (AIS) since 2014. After the introduction of endoscopic decompression, instead of treating the spondylolysis itself, treatment can specifically target the refractory radicular pain developed during the degeneration progress without devastating the peripheral soft tissue. However, we noticed that endoscopic transforaminal decompression seems to be less effective in AIS compared to other types of degenerative spondylolisthesis. Thus, we came up with a novel craniocaudal interlaminar approach, utilizing the proximal adjacent interlaminar space to perform bilateral decompression and observed the pathoanatomy of pars defect directly and tried to identify the cause of decompression failure.

**Methods:**

From January 2022 to June 2022, 13 patients with AIS underwent endoscopic decompression via the endoscopic craniocaudal interlaminar approach and were followed up for at least 6 months. Visual Analogue Scale, Oswestry Disability Index and MacNab scores were recorded to monitor patients’ clinical recovery. All endoscopic procedures were recorded and reviewed to illustrate the pathoanatomy.

**Results:**

Four patients required minor revision via the same technique. One of them required it due to incomplete isthmic spur resection, two due to neglected disc protrusion, and the other due to root subpedicular kinking in higher grade anterolisthesis. All patients’ clinical condition improved significantly subsequently. After reviewing the endoscopic video, we have observed that the hook-like, ragged spur originating from the isthmic defect extends beyond the region around the foramen. Instead, it extends proximally into the adjacent lateral recess, resulting in impingement along the fracture edge above the index foramen and, in some cases, even in the extraforaminal area.

**Conclusions:**

The broad spanning isthmic spur extending to the proximal adjacent lateral recess might be the reason why the transforaminal approach yielded less satisfactory results due to the incomplete decompression result from approach related restriction. Our study demonstrated an optimistic outcome by applying decompression from the upper level. Therefore, we propose that the craniocaudal interlaminar approach might be a better route for decompression in adult isthmic spondylolisthesis.

## Background

Spondylolysis is generally considered an acquired defect or fracture at the pars interarticularis due to repetitive extension stress during the early stage of life [1–3]. The defect compromises the continuity of posterior ligamentous components of the spine that several reviews have reported nearly eighty percent of patients with bilateral pars defects developed adult isthmic spondylolisthesis (AIS) [4, 5]. Decompression, decompression with instrumentation, or spinal fusion has been considered as a reasonable treatment option for AIS although the NASS guideline cannot recommend or kick against any of the surgical treatment options due to controversial results [6–10]. The progression of anterior slippage, which occurred majorly during the early stage of life, will eventually stop and a stable condition will be reached whether the pars defect achieved bony or fibrous union [4, 11, 12]. Most patients can become pain-free with appropriate conservative management after reaching this stage [13]. However, the distraction stress at the defect during slippage results in fibrocartilaginous scar formation, bone spur ingrowth, and the accelerated degeneration or herniation of the intervertebral disc, which leads to the diminished height of the neural foramen, together, can cause foraminal stenosis radiculopathy [14].

Compared to other forms of degenerative spondylolisthesis, radicular pain from AIS can develop a few years earlier in life. Instead, of treating the pars defect itself, decompression alone could be effective as a salvage procedure for the younger patients to postpone fusion surgery or a symptom relief procedure for the elders who cannot endure spine fusion. With the development of endoscopic spine surgery, more and more endoscopic decompression procedures including the transforaminal and interlaminar approaches were adapted to treat AIS patients who suffered from radiculopathy [15–19]. Endoscopic decompression shares the benefits of precision and peripheral soft tissue sparing, theoretically decreasing the risk of postoperative instability [20]. It can be grossly categorized into the interlaminar approach (ILA) and the transforaminal approach (TFA) [21]. In foraminal stenosis, the transforaminal approach seems to be more rational due to its direct access to the foramen where the pathologic structures are located. However, based on our experience, transforaminal decompression seems to be less effective in adult isthmic spondylolisthesis than other types of degenerative spondylolisthesis. Moreover, a prolonged spanning pattern of the isthmic ragged spur from the lateral border of the lamina to the very medial (and even proximal) border of the lamina has been observed, which is too distant and difficult to reach via the transforaminal approach [22, 23]. This might result in incomplete decompression due to the natural anatomical limitation.

To solve this issue, we reported our novel endoscopic craniocaudal interlaminar approach (CIA), which utilizes the proximal adjacent interlaminar space to observe the lesion directly from above, and strive for a complete decompression in AIS radiculopathy. We also reported the pathoanatomy of AIS we have observed during the operation, compared the pros and cons of different approaches in this situation, and discussed why the CIA might be reasonable and effective.

## Material and methods

### Patient selection and clinical evaluation

Between January 2022 and June 2022, we retrospectively recruited thirteen patients with AIS-related radiculopathy who underwent endoscopic craniocaudal interlaminar decompression surgery for foraminal stenosis. The patients were followed up for at least 6 months. Patients at a higher risk of postoperative instability, such as those with high-grade spondylolisthesis (greater than Meyerding grade II) and vertebra endplate microfractures, were excluded from the study. Additionally, patients with multilevel stenosis and those with simple lumbar disc herniations lacking significant pars defect degeneration on preoperative radiographs were also excluded. Visual Analogue Scale (VAS), Oswestry Disability Index (ODI) and MacNab scores were used to assess clinical recovery and patients’ satisfaction. Statistical assessments were performed to compare the preoperative, postoperative (within 1 week), and 6-month follow-up outcomes. The procedures were conducted by a single experienced surgeon and were recorded using an endoscopic system. The recorded videos were independently reviewed by three orthopedic surgeons to document the observed pathoanatomy during the operation and compare it with the preoperative images.

### Statistical analysis

Statistical analysis was performed between the pre- and postoperative clinical results using Wilcoxon signed-rank test and paired t-test on SPSS version 20 (IBM). Statistical significance was defined at *P* < 0.05.

### Surgical approaches

**Fig. 1:**
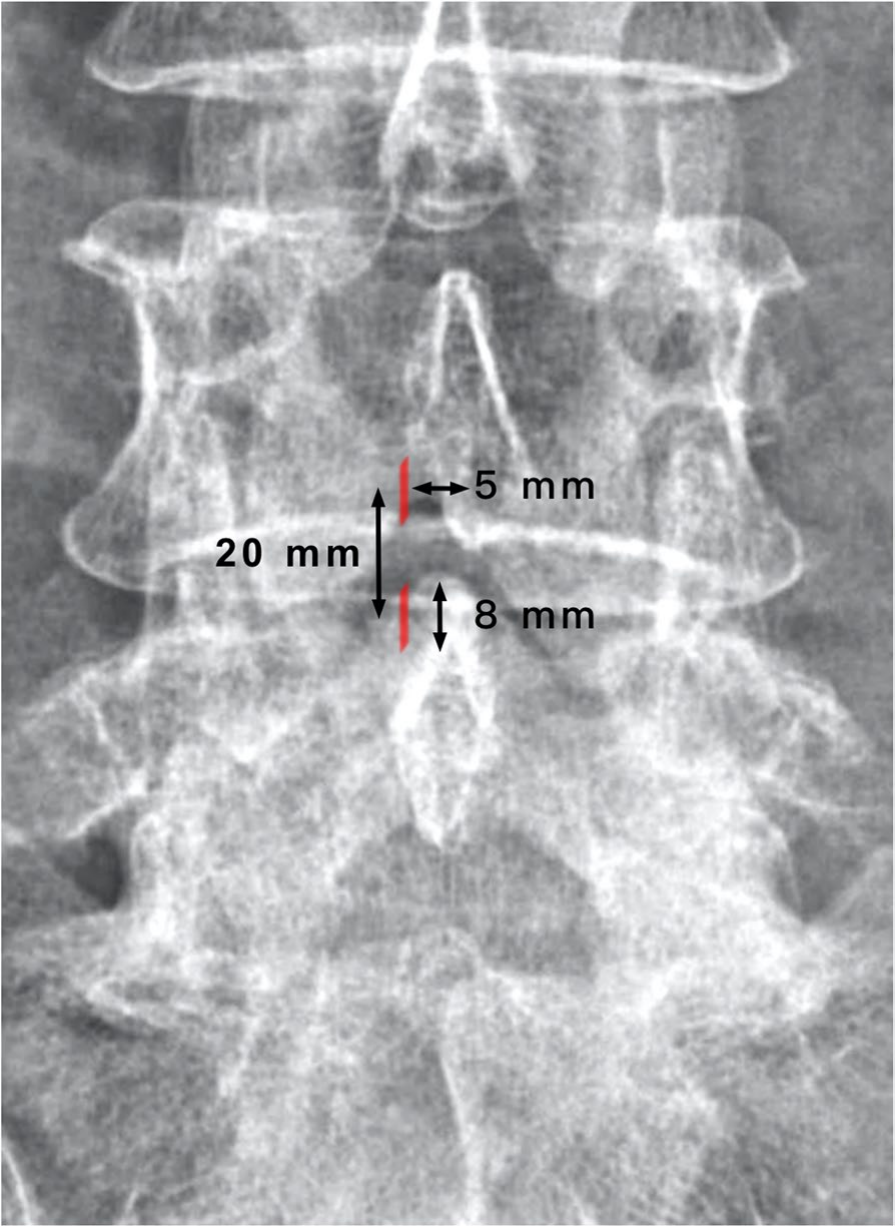
The portal incision is located on the upper border of the interlaminar space, 5 mm lateral to the spinous process, and the instrumental incision is about 2 cm distal to the portal incision

The patient was placed in the prone position on a radiolucent table with a soft cushion that was well padded at the bony prominences of the body after general or spinal anesthesia according to the patient’s condition. Level confirmation and marking are conducted under fluoroscopic guidance before the incision is performed. The interlaminar space between the target vertebra and the vertebra above it is utilized. The first incision for the scope portal is located on the upper border of the interlaminar space, 5 mm lateral to the spinous process, and the second incision for the instrumental portal is about 2 cm distal to the first incision (Fig. 1). The muscle fascia perpendicular to the skin is incised to prevent the obstruction of water flow during surgery. A smooth periosteal elevator is used to detach and shift the paraspinal muscle and other soft tissue from the interlaminar space to the lateral side. After successfully introducing the endoscope into the interlaminar space, shavers, and radiofrequency wands are used to clear the rugged muscle and debris within it. To create a sufficient working space for instruments, wedge resection of the spinous process base and lamina border of the upper and index vertebra are sometimes needed.

**Fig. 2:**
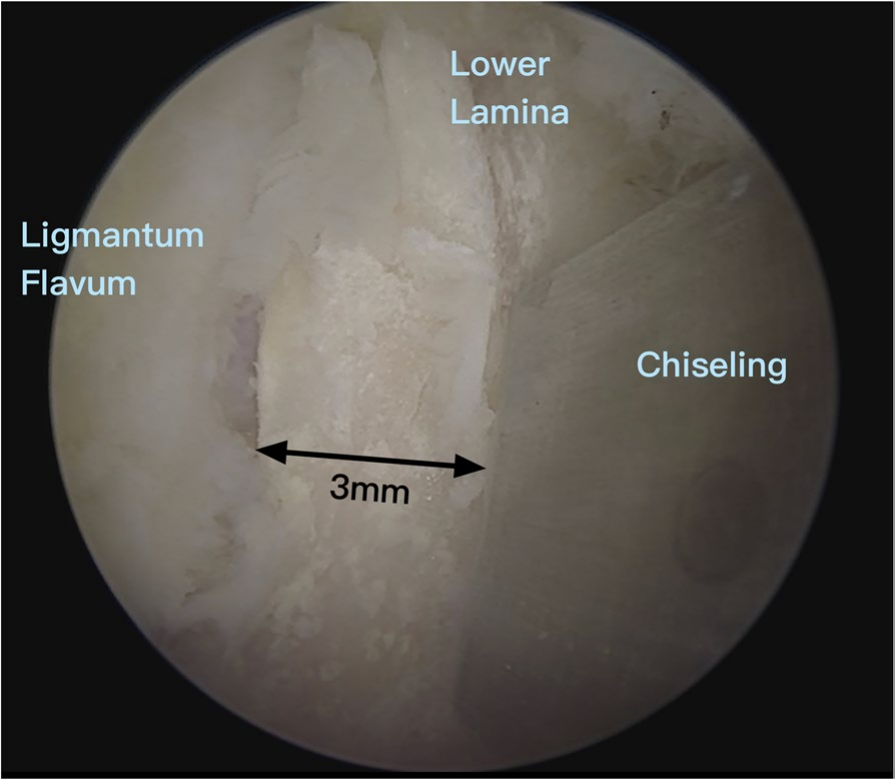
A cranial laminotomy of about 3 mm is performed using a chisel

**Fig. 3:**
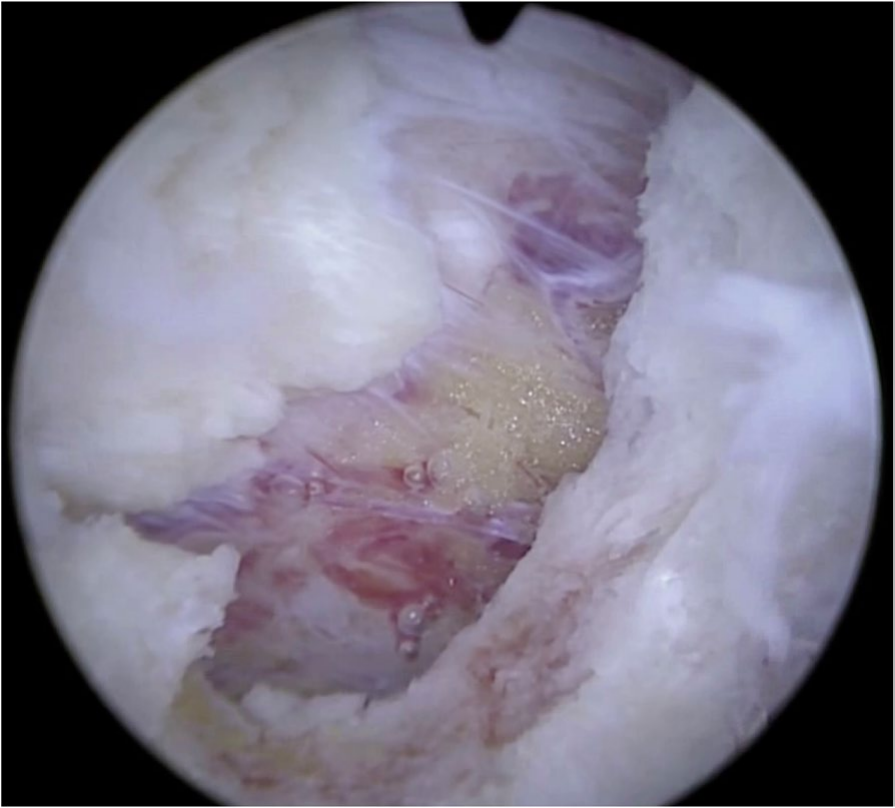
The insertion of the deep ligamentum flavum can be taken down along with the resected lamina edge, exposing the epidural region

**Fig. 4:**
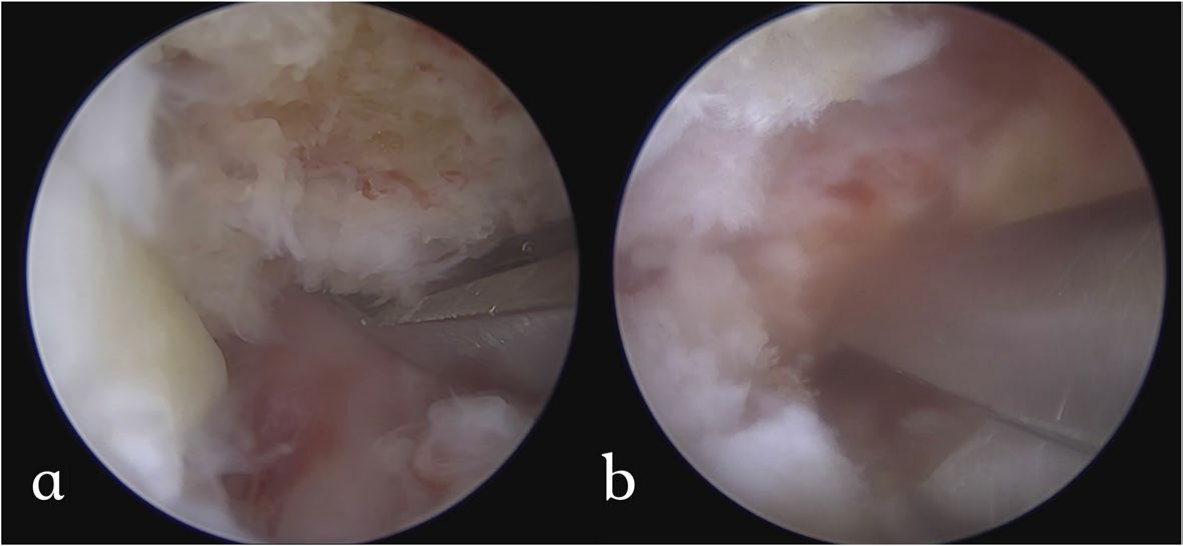
The laminotomy of the Gill fragment was continued laterally with a Kerrison punch bit by bit until the pars defect was reached. **a** Laminotomy and adhesiolysis were done at the contralateral isthmic defect; **b** and the ipsilateral isthmic defect

After a smooth instrument workflow is confirmed, the superficial ligamentum flavum is removed for better visualization of the upper border of the index lamina (Gill fragment). A cranial laminotomy of about 3 mm is performed with a chisel or osteotome (Fig. 2). A high-speed burr can be used to create a starting point first for laminotomy due to the hypermobility of the Gill fragment. The insertion of the deep ligamentum flavum can be taken down along with the resected lamina edge, exposing the epidural region (Fig. 3). The laminotomy of the Gill fragment was performed bilaterally and extended laterally using a Kerrison punch. This was done incrementally until the pars defect was reached (Fig. 4). However, during the process of tracing the cranial edge of the Gill fragment to the proximal lamina remnant, it is advised to exercise extra caution due to the narrow field of view offered by endoscopy. Additionally, gently pushing the Gill fragment with a surgical instrument can assist in determining its orientation. In higher-grade spondylolisthesis, the proximal lamina remnant can usually be discovered after passing the fibrocartilage adhesion gap between the defect. However, when attempting to access the contralateral side of the lamina remnant, one can easily get lost in the dorsal muscle layer instead of the correct lamina surface of the lower vertebra due to the height difference between the Gill fragment and the remnant proximal lamina. If the L4–5 facet joint is located at the same level as the Gill fragment due to the ventral prolapse of the L5 vertebra, the gap between the inferior articular process of the upper vertebra and the Gill fragment can be misinterpreted as an isthmic defect. In lower-grade spondylolisthesis, the proximal lamina remnant can be concealed beneath the Gill fragment. To locate the defect precisely, conventional distal laminotomy of the upper vertebra can be performed first, progressing laterally and distally to reach the facet joint and pedicle of the index vertebra. With sufficient adhesiolysis around the pedicle and lateral recess, the proximal lamina remnant can be discovered as part of the foramen roof of the index vertebra.

**Fig. 5:**
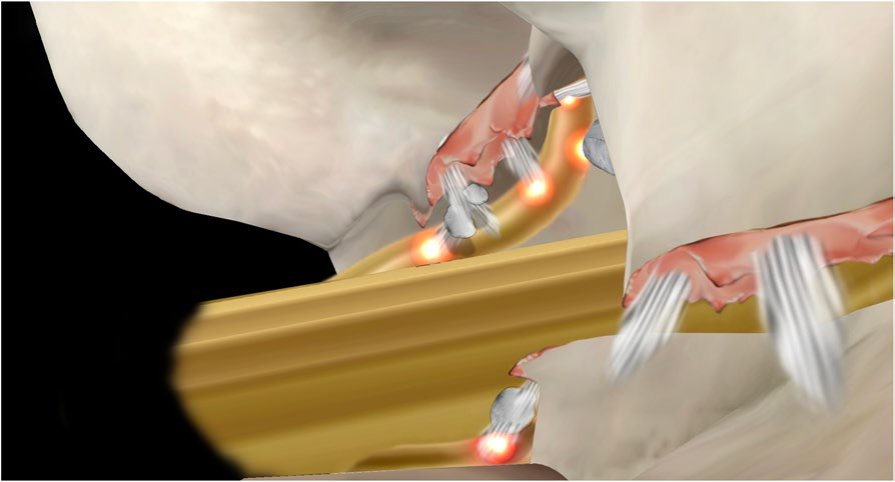
The four common pathoanatomy in AIS radiculopathy

**Fig. 6:**
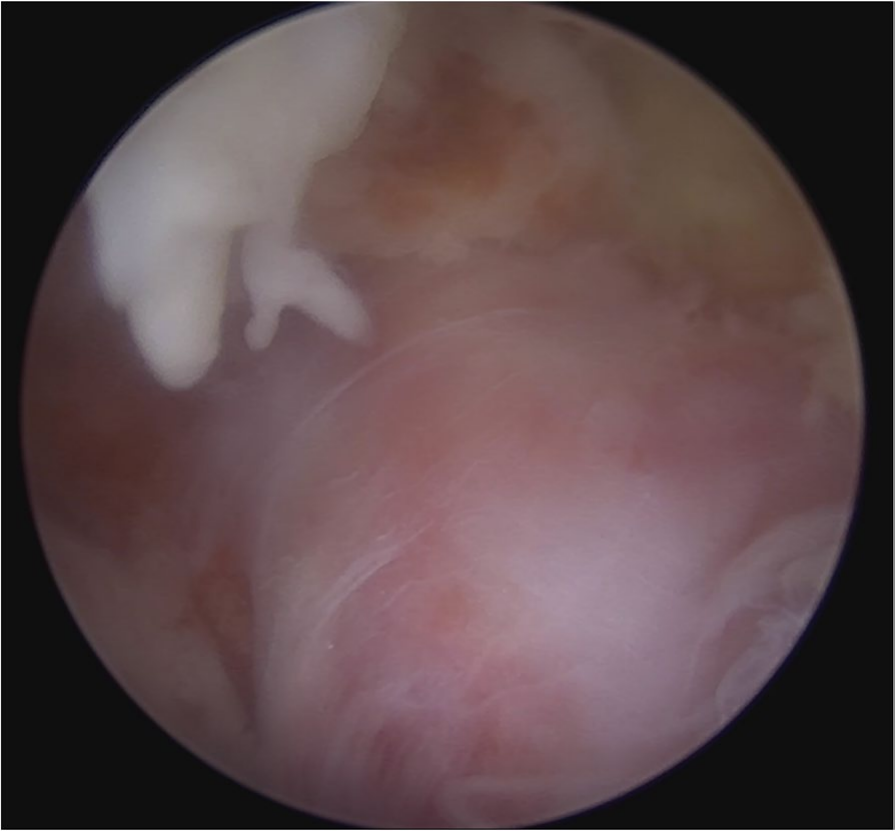
After sufficient adhesiolysis of the pars defect, the inflammatory nerve root underneath can be exposed

**Fig. 7:**
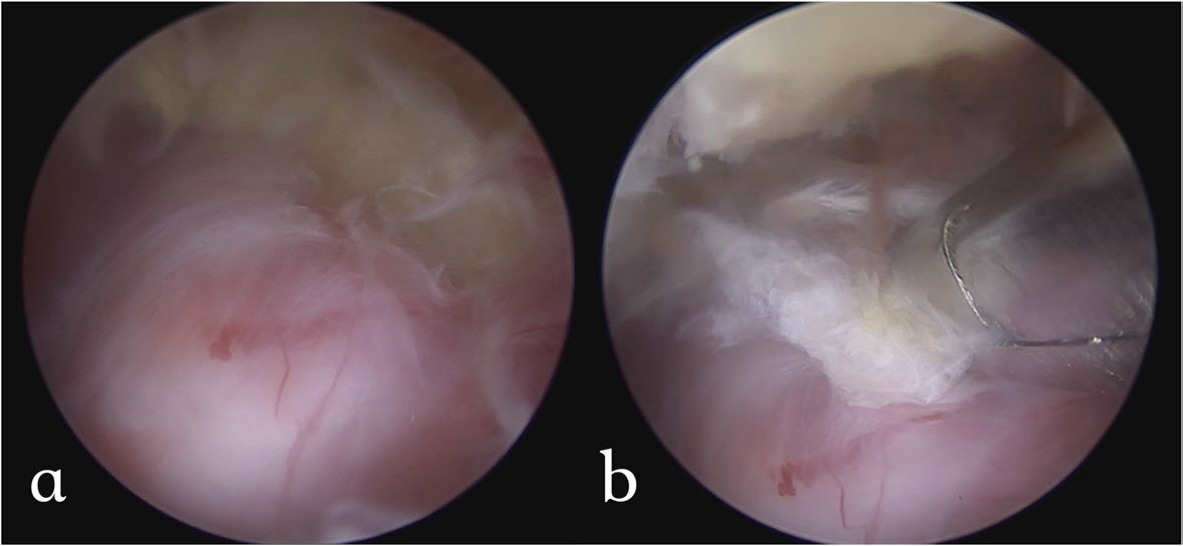
**a** The extruded disc owing to lumbar lordosis and anterolisthesis can be easily neglected, making the pathologic disc disguise under the exiting root as the floor; **b** The extracted disc materials after annulectomy

**Fig. 8:**
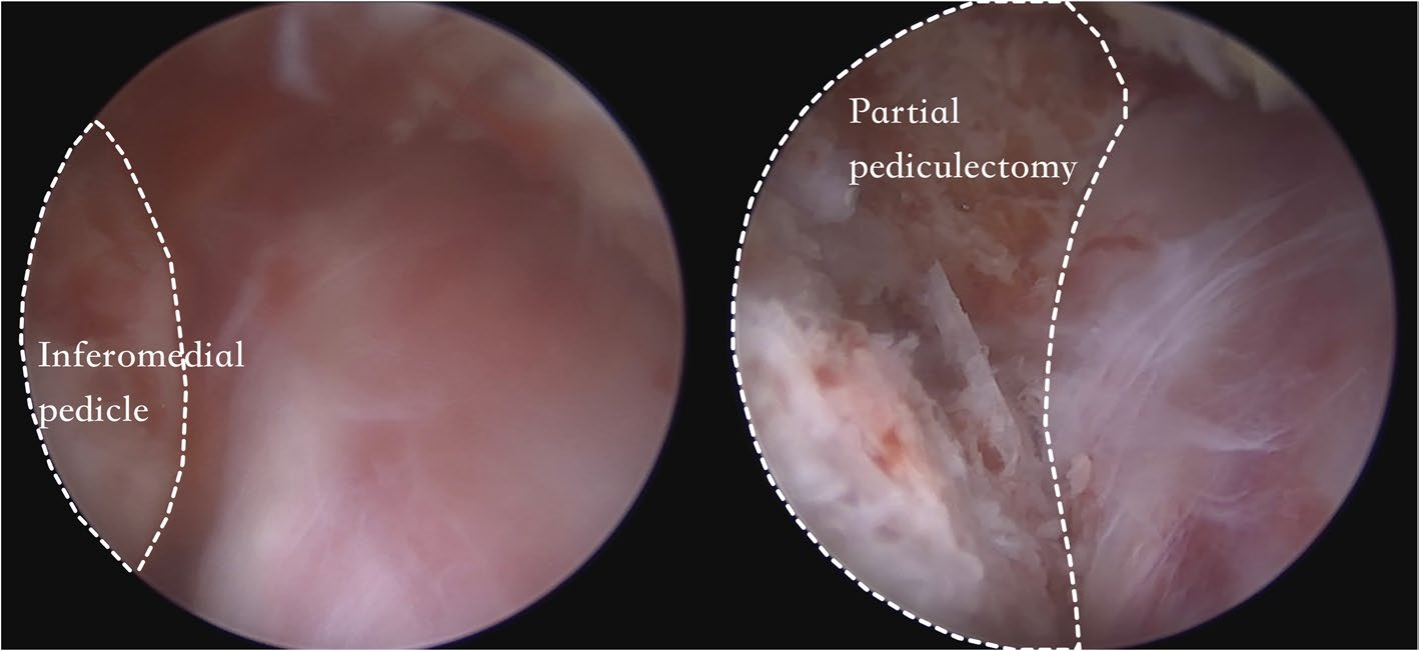
Inferomedial partial pediculetomy can be done to relieve the exiting root tension from the subpedicular kinking caused by the anterolisthesis

There are four lesions that should be addressed in the AIS foraminal stenosis: the isthmic spur overlying the exiting root from lateral recess to the foramen, fibrocartilage adhesion and a loose body within the canal, the foraminal ligament, and the buckled and/or extruded foraminal disc (Fig. 5). After successful identification of the pars defect, the isthmic spur from both the Gill fragment and the proximal lamina remnant were removed bilaterally using an ergonomic and centrally bounded approach at a cranial angle. The Gill fragment can be shifted more distally and posteriorly via the instrument or endoscope after sufficient adhesiolysis, uncovering the inflammatory region underneath (Fig. 6). The fibrocartilage adhesion and loose body within the defect or canal should all be addressed completely. Due to the trajectory similarity of the approach and fracture direction, the root can be traced to the extraforaminal area along the distal pedicle margin, although the extraforaminal portion of the pars defect has less influence on the radiculopathy because of the ventral projection of the nerve root. We suggest that the decompression depth be set at a point where we can identify the free adipose tissue at the cranial side of the exiting root, which represents reaching the accessory process of the transverse process. The last target: the protruded or extruded disc can be easily neglected due to the lumbar lordosis and anterolisthesis, making the pathologic disc disguise under the exiting root as the floor (Fig. 7). We should strive to reveal the deformed annulus and endplate spur underneath and remove it as completely as possible. Even if the foramen seems to have sufficient space for the nerve root, to prevent dynamic compression in AIS when the patient stands, a partial diskectomy is recommended if a protruded disc is identified on preoperative MRI. After the decompression, we also suggest inferomedial partial pediculetomy to relieve the exiting root tension from the subpedicular kinking caused by the anterolisthesis (Fig. 8). The ideal full decompression should make the root drift from the vertebral body and recover the natural root pulsation. After the hemostasis, a drain is placed in the epidural space and the wound is closed using 4–0 nylon sutures.

## Results

Thirteen patients (seven males and six females) with a mean age of 61.1 ± 12.9 (34–81) years were included in this study. The index level of spondylolysis occurred at L4 in 3 cases and L5 in 10 cases. The VAS, ODI, and MacNab scores differed significantly after surgery (Table 1) There were 4 cases of revision via the same technique, one due to incomplete isthmic spur resection, two due to neglected disc protrusion, and the other due to root subpedicular kinking in higher grade anterolisthesis. The symptoms all relieved after the revision surgery.

**Table 1:** Statistical analysis of the clinical satisfaction

	Pre-OP	Post-OP	Post 6 m	Last-FU	*P*-value*	
					Pre-PO	Pre-Last FU
VAS-Back	7.1 ± 0.6	3.6 ± 1.3	2.5 ± 1.6	2.7 ± 1.9	< 0.001	< 0.001
VAS-Leg	7.2 ± 1.6	3.3 ± 1.0	1.9 ± 1.8	2.1 ± 1.2	< 0.001	< 0.001
ODI	43.4 ± 4.2	25.5 ± 7.0	20.7 ± 7.4	19.0 ± 7.4	< 0.001	< 0.001

^*^P-value, by Wilcoxon signed-rank test

A total of seventeen video clips were reviewed. All cases revealed proximal adjacent lateral recess spur and foraminal spur, and four cases revealed extraforaminal spur. Four cases revealed foraminal disc extrusion and one case revealed extraforaminal disc extrusion. The upper-level lateral recess stenosis were involved in every cases, resulting from the extended isthmic ragged spur, fibrocartilage adhesion, and loose body (Table 2).

**Table 2:** Patient’s demographic data, pathoanatomy, and clinical outcome

**Patient no**	**Sex**	**Age**	**AIS grade**	**Decompression side**	**Isthmic spur**	**Preoperative**		**Postoperative**		**MacNab**	**Revision**
						**VAS**	**ODI**	**VAS**	**ODI**		
1	M	34	L5 Gr I	Right	Lateral recess Foramen	8	45	0	12	Excellent	-
2	M	60	L5 Gr I	Bilateral	Lateral recess Foramen	8	45	1	19	Excellent	-
3	M	71	L5 Gr I	Left	Lateral recess Foramen Extraforamen	8	48	6	42	Fair	Incomplete isthmic spur decompression
4	F	57	L5 Gr I	Bilateral	Lateral recess Foramen	2	42	0	16	Good	-
5	M	67	L5 Gr II	Bilateral	Lateral recess Foramen Extraforamen	8	48	0	18	Good	Nerve root subpedicular kinking due to higher anterolisthesis
6	F	39	L5 Gr I	Bilateral	Lateral recess Foramen	5	35	1	17	Excellent	Neglected protruded foraminal disc
7	F	72	L5 Gr II	Bilateral	Lateral recess Foramen Extraforamen	8	42	3	17	Good	-
8	M	56	L5 Gr II	Bilateral	Lateral recess Foramen	7	42	3	16	Excellent	-
9	F	65	L5 Gr I	Bilateral	Lateral recess Foramen Extraforamen	8	38	3	16	Good	-
10	M	68	L4 Gr I	Bilateral	Lateral recess Foramen	7	46	0	16	Good	-
11	F	60	L4 Gr II	Bilateral	Lateral recess Foramen	7	48	0	14	Excellent	-
12	F	64	L5 Gr I	Bilateral	Lateral recess Foramen	7	46	0	18	Good	Neglected protruded foraminal disc
13	M	81	L4 Gr I	Bilateral	Lateral recess Foramen	7	39	2	15	Excellent	

## Discussion and conclusions

### Pathoanatomy

**Fig. 9:**
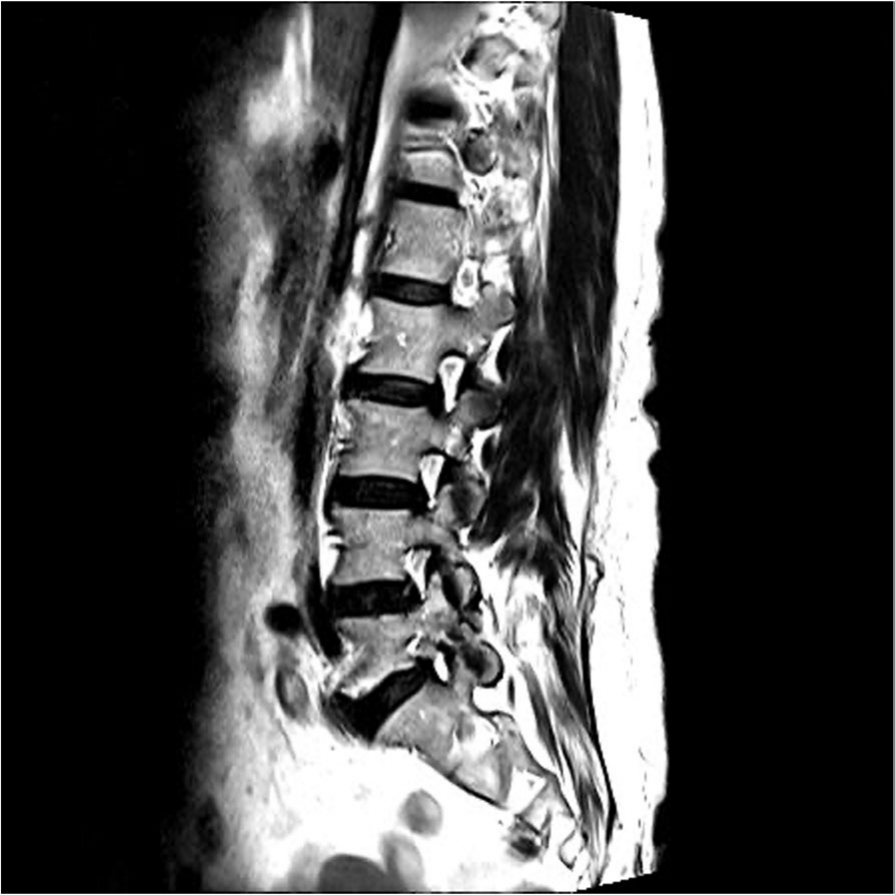
The sagittal view of MRI were effective in revealing the foraminal stenosis

**Fig. 10:**
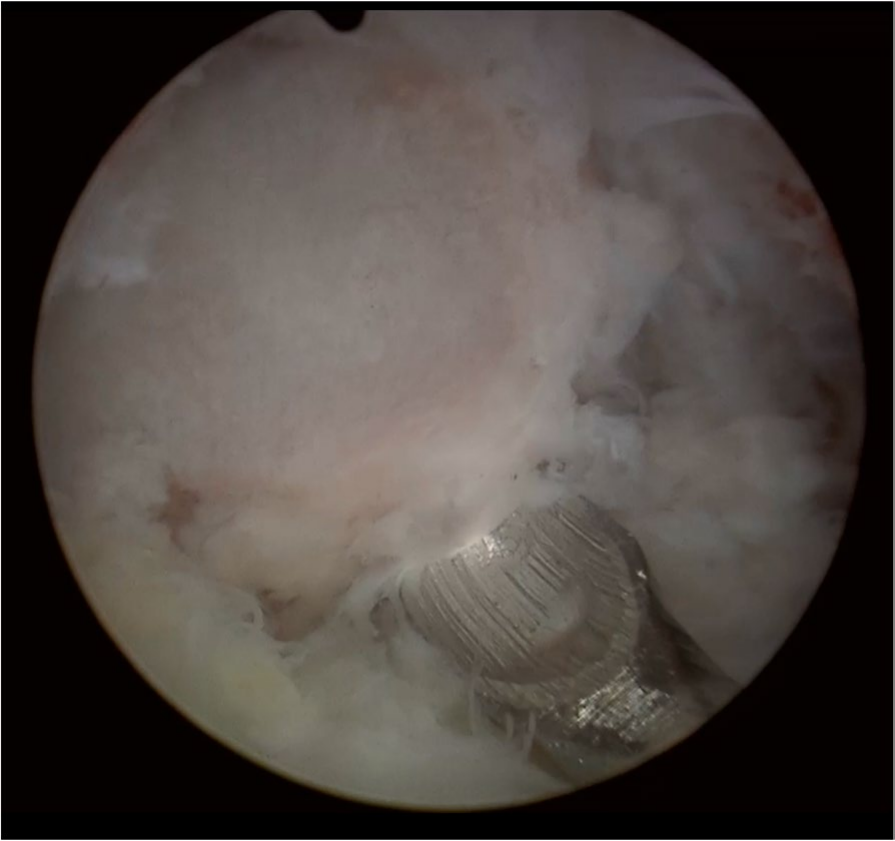
The isthmic ragged spur grows obliquely like a hook, spanning from the adjacent proximal lateral recess to the index foramen

**Fig. 11:**
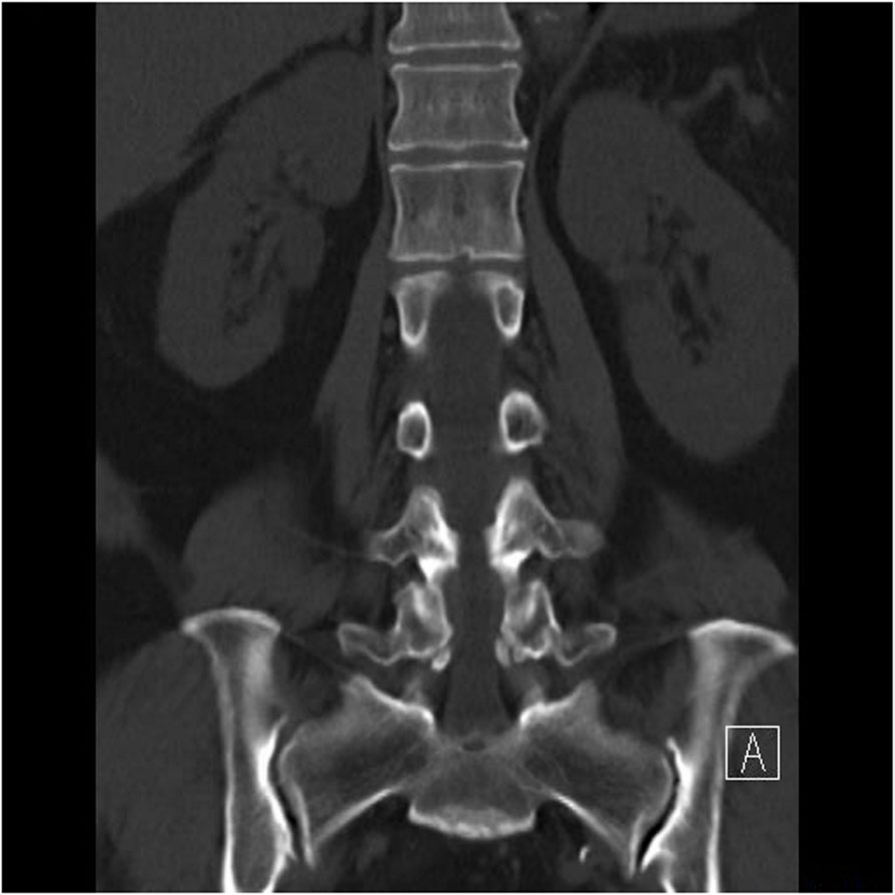
The coronal view of CT effectively reveals the cranial lateral recess spur configuration

According to the integrity of the pars defect in AIS, the facets and lamina are spared from the weight transmission stress, resulting in foraminal stenosis radiculopathy rather than lateral recess stenosis radiculopathy in other kinds of degenerative spondylolisthesis [14]. The foraminal spur can be revealed under the sagittal view of Magnetic Resonance Imaging (MRI) (Fig. 9). However, we have noticed that even when patients underwent proper endoscopic decompression via the transforaminal approach, the prevalence of residual pain and the recurrence rate appear to be slightly higher in AIS patients than in patients with other forms of degenerative neuroforaminal stenosis. After reviewing the images and intraoperative endoscopic videos, we have observed that the hook-like isthmic ragged spur, which has been mentioned in previous literature, exhibits a unique growth pattern [22, 23]. Specifically, the spur mostly extends beyond the isthmic defect and grows proximally into the proximal adjacent lateral recess (Fig. 10). The ragged spur spans the isthmic defect from the lateral border of the lamina to the medial and, cranial parts of the same (Fig. 5). This extension causes nerve root impingement, especially at the narrowest part of the foramen where it is adjunct to the upper-level lateral recess, as spondylolisthesis progresses. Although lateral recess stenosis has been previously illustrated, it is seldom mentioned as a critical factor during endoscopic isthmic decompression [24]. As a matter of fact, the irritation of the exiting root begins already at the lateral recess of the upper level along the foramen and the extraforaminal region of the index level. We also found that the coronal view of CT effectively reveals the recess spur configuration (Fig. 11). Therefore, we hypothesize that this could be the factor why TFA revealed less satisfactory results in our experience: Although TFA is excellent in addressing the extraforaminal and foraminal lesions, the lateral recess of the upper level can be neglected.

### Surgical decision

**Fig. 12:**
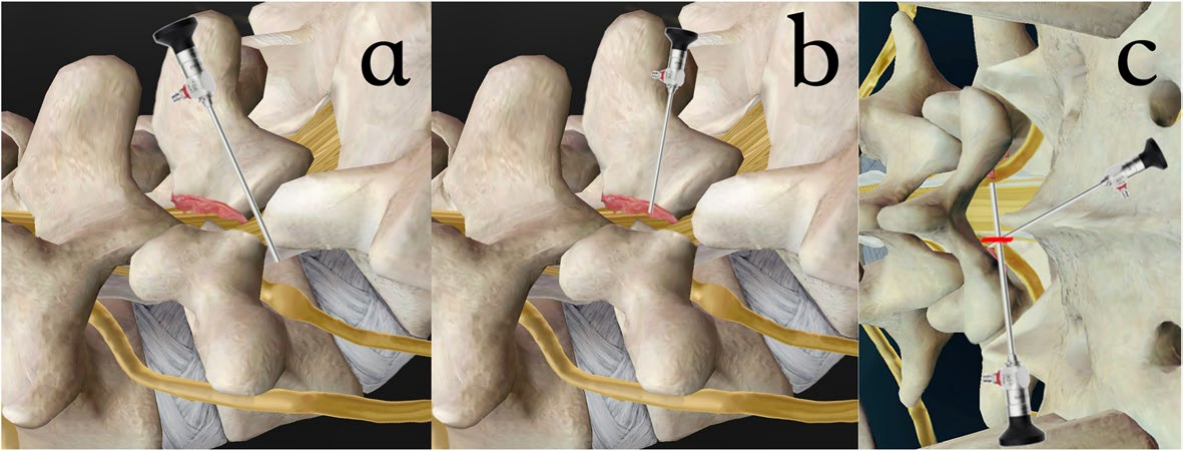
**a** The transforaminal approach; **b** The pars *in-situ* approach; **c** The index level interlaminar approach

However, the transforaminal approach was adapted in most previous studies, even though the index vertebra was often L5 (the vertebra with the longest intervertebral foramen and with a high-blocking iliac crest from below) [15–18]. To overcome these anatomical obstacles, the trajectory of the transforaminal approach should become more horizontal- and caudal-aiming, at the same time making the proximal adjacent lateral recess less accessible. From the aspect of the transforaminal approach, the lateral laminar remnant spur within the extraforaminal region and foramen can be reached; however, the proximal and medial laminar remnant spurs will be in an eccentric direction and even be blocked by the pedicle, making resection less ergonomic (Fig. 12a). In patients with higher-grade anterolisthesis, the Gill fragment spur can be too dorsal to reach. To overcome this difficulty, we have tried modifying the transforaminal approach to a pars *in-situ* approach, landing the scope and instrument directly on the pars defect (Fig. 12b). This approach successfully achieved the goal of simultaneous resection of the spur from the proximal lamina remnant and Gill fragment; however, massive bleeding and the loss of direction are commonly encountered due to the thick paraspinal muscle and abundant blood supply in this area. Sharing the same disadvantages as the transforaminal approach, in patients with bilateral symptoms, a bilateral approach is mandatory, which prolongs surgery and increases the anesthesia time.

In foraminal stenosis radiculopathy, the conventional interlaminar approach seems to be impractical while the main pathology is scattered mostly along the bilateral neuroforamen, making the strength of the interlaminar approach such as sublaminar and index lateral recess decompression ineffective. Given that L5–S1 provides the largest interlaminar space with hypermobility of the Gill fragment for instrument manipulation, a much wider distal laminotomy is still needed to gain access into the bilateral neuroforamen and the proximal adjacent lateral recess (Fig. 12c). However, Kaneko et al. reported an excellent short-term result of utilization of the interlaminar approach in AIS patients, with the decision being based on the far migrated disc of the patients and the concern of difficulties that could be encountered while gaining access through the longest L5 foramen and high iliac crest obstruction in the transforaminal approach [19].

**Fig. 13:**
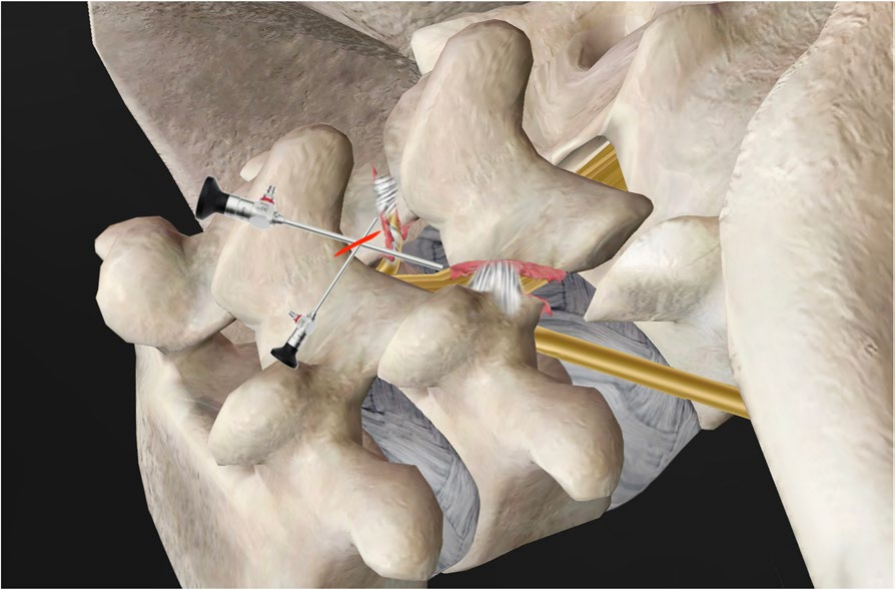
The craniocaudal interlaminar approach utilizing the upper level interlaminar space can address bilateral isthmic spur ergonomically and simultaneously, fulfilling unilateral laminotomy bilateral decompression

Eventually, we came up with the CIA. Utilizing the proximal adjacent interlaminar space and approach via the craniocaudal trajectory, we can reach the bilateral main pathology with less difficulty. With the endoscopic view from the upper level, bilateral isthmic spur from the Gill fragment and proximal lamina remnant can be addressed ergonomically and simultaneously, just like performing the conventional distal laminectomy in open surgery, doing it twice, not to mention that the lateral recess decompression, inherently, is one of the greatest advantages of the interlaminar approach, also fulfilling unilateral laminotomy bilateral decompression (Fig. 13). A similar concept was previously introduced by Sairyo et al., who utilized microendoscopic technique to address bilateral isthmic spur from the upper adjacent level [25]. Yamashita et al. later reported a case in which this technique was combined with a transforaminal approach to treat an AIS patient with a far-lateral disc [26]. In patients with unilateral symptoms, the CIA can be approached from the contralateral side to permit deeper access to the extraforaminal region. In patients with bilateral symptoms, the left-side unilateral approach can address right-side lesions from the lateral recess to the extraforaminal region while the left side can be reached, at least to the exit of the foramen, depending on the grade of anterolisthesis. According to what we have observed from the endoscopic video clips, most of the pathoanatomy is located at the proximal adjacent lateral recess and the foraminal region, and CIA can address the lesion in this area ergonomically compared with the transforaminal approach which is good for decompression at the extraforaminal region and the foramen. Thus, we proposed CIA for radicular decompression in AIS. A summary of the current state of research on endoscopic approaches to AIS is presented in Table 3.

**Table 3:** A summary of current literatures regarding endoscopic approaches to AIS

**Study**	**Year**	**Approach**	**Patient Number**	**Age**	**Scenario**	**Outcome**
Knight et al.	2003	Uniportal Transforaminal Approach	24	42.4 y/o (22–72)	• Back pain, buttock pain with or without leg pain • L45, L5S1 Meyerding grade I–III	• Postoperative 2 years, VAS demonstrated that 5 (21%) were pain free, 15 (63%) had 50% change in VAS, 3 were poor and 3 were worse. • 2 patients underwent revision spinal fusion.
Madhavan et al.	2016	Uniportal Transforaminal Approach	1	31 y/o	• Leg pain and mild back pain for more than 1 year. Leg pain worse than the back pain. Progressively worsened to unable to walk more than 10 feet. • L5S1 Meyerding grade I • Left paracentral disc	• At 2 years after surgery, he continued to be free from radicular and back pain, with a sustained ODI score of 8. • Preoperative VAS were 8–9 of 10, postoperative VAS scores were 1–2 of 10. Preoperative ODI was 74, and was reduced to 8.
Yeung et al.	2018	Uniportal Transforaminal Approach	5	56.6 y/o (27–71)	• Sciatica > back pain • L5S1 low grade spondylolisthesis	• Postoperative 2 years comparison: VAS were 2(7), 4(7), 1(4), 2(6), and 0(4); ODI were 32(48), 19(32), 28(42), 14(24), and 12(17); Macnab showed 3 excellent and 2 good results • No patient underwent spinal fusion
Liu et al.	2020	Uniportal Transforaminal Approach	2	56 y/o	**Case 1** • Left leg pain with left foot drop. • L5S1 Meyerding grade II without spinal instability. • Treated with left TFELD. • 2 years after, right leg pain along the L5 dermatome. • Treated with right TFELD.	**Case 1** • 5 years after left TFELD, 2 years after the right TFELD, VAS scores were 0 for the right leg and 4 for the left leg and back; ODI was 0. VAS were 7 for left leg and back, and ODI was 56, preoperatively.
				63 y/o	**Case 2** • Chronic low back pain and right leg pain with tingling and numbness along the right L5 dermatome for years. • L5S1 Meyerding grade I without spinal instability.	**Case 2** • Postoperative 1 year comparison: VAS was 3(6–8) and ODI was 30(40).
Kaneko et al.	2021	Uniportal Interlaminar Approach	2	55 y/o	**Case 1** • Left leg and low back pain for 2 months. Left SLRT 50°, no apparent muscle weakness was observed. • L5S1 Meyerding grade I • Upward-migrated L5S1 disc and compressed L5 nerve root	**Case 1** • Postoperative 1 year comparison: ODI was 4(36), RDQ24 was 0(12), and NRS was 0(10).
				51 y/o	**Case 2** • Right leg pain without low back pain. Bilateral SLRT 30°, no apparent muscle weakness was observed. • L5S1 Meyerding grade I • Intracanal L5S1 disc	**Case 2** • Postoperative 1 year comparison: ODI was 2(18), RDQ24 was 0(7), and was NRS 0(8).
Wu et al.	2023	Biportal Craniocaudal Interlaminar Approach	13	61.1 y/o (34–81)	• Back pain, buttock pain with leg pain • L45, L5S1 Meyerding grade I–II	• Postoperative 6 months, VAS, ODI, and MacNab demonstrated significant improvement. • 4 cases underwent revision via the same technique, one due to incomplete isthmic spur resection, two due to neglected disc protrusion, and the other due to root subpedicular kinking in higher grade anterolisthesis. The symptoms all relieved after the revision surgery. • No patients required revision spinal fusion.

From our experience, endoscopic decompression alone has great benefits in younger patients whose spinal fusion can be too early for only radicular symptoms in senile patients for who fusion surgery can constitute a huge burden and a major stressor. From our experiences, grade I or grade II AIS patients with a disc height of less than 20% have a lower risk of ventral slip progression. The presence of fibrosis around the disc and the stabilization of the anterior vertebral osteophyte usually permits less than 4 mm of ventral prolapse in flexion and extension comparison views. In patients with significant dynamic instability such as a ventral translation of more than 4 mm and an angle difference of more than 10 degrees in dynamic views, and in patients with a residual disc height of more than 50%, there could be a higher probability of recurrent disc rupture and further collapse. Decompression with fusion might be a more optimistic treatment in this group of patients.

## Limitations

Endoscopic decompression surgery via CIA continues to demonstrate promising results in radiculopathy in AIS; however, there are still potential complications we should beware of [27]. Although the CIA utilized the interlaminar space just like the conventional interlaminar approach, advancing the endoscope and instrument to the caudal foramen, and even the extraforaminal area, can be a totally different horizon. Double-checking with fluoroscopy is always suggested when the anatomy under the scope becomes unrecognized. A misinterpreted anatomy during laminotomy can result in iatrogenic fractures, even in the hands of an experienced surgeon. Since this technique utilizes the proximal adjacent interlaminar space to approach the lysis, accessing the adjacent segment in the future may become more difficult due to adhesions or other changes in the anatomy caused by the approach. This surgical technique offers the advantage of achieving bilateral decompression through a single approach. However, due to anatomical limitations, it can only address left-side lesions up to the foraminal region, while right-side lesions can be addressed from the lateral recess to the extraforaminal region. Therefore, if a left extraforaminal far-lateral extruded disc is present, an alternative approach may be required. Although the short-term outcomes of the CIA have been promising, the study only followed up with thirteen patients for 6 months. Larger sample sizes and longer follow-up periods will be required to validate these results.

## Conclusions

Based on our experience, after mastering the pearls and pitfalls of this technique, endoscopic CIA is highly effective in relieving a patient’s refractory radicular pain following failed conservative treatment. The goal of endoscopic foraminal decompression is not to treat spondylolysis or spondylolisthesis; rather, it is one of several personalized alternatives to alleviate the painful transitional stage. However, the ideal indication for endoscopic decompression still requires further investigation.

## Data Availability

The datasets used and/or analysed during the current study are available from the corresponding author on reasonable request.

## Abbreviations

AIS: Adult isthmic spondylolisthesis
VAS: Visual analogue scale
ODI: Oswestry disability index
ILA: Interlaminar approach
TFA: Transforaminal approach
MRI: Magnetic resonance imaging
CT: Computerized tomography
